# Malrotation in an Adult Patient With Pneumoperitoneum Following Nissen-Sleeve Gastrectomy: A Case Report

**DOI:** 10.7759/cureus.53672

**Published:** 2024-02-05

**Authors:** Abdulmenem Y Abualsel, Abdullah A Bashandi, Ghadeer E AlMohamed Ali

**Affiliations:** 1 Department of General Surgery, King Hamad University Hospital, Muharraq, BHR; 2 Department of General Surgery, Salmaniya Medical Complex, Manama, BHR

**Keywords:** diagnostic laparoscopy, post-sleeve gastrectomy, non-rotation, massive pneumoperitoneum, incidental diagnosis, adult intestinal malrotation

## Abstract

Intestinal malrotation is typically encountered in the first year of life and is rarely seen in adult populations. Herein, we present the case of a 48-year-old woman with a surgical history of laparoscopic Nissen-sleeve gastrectomy before 11 months who was referred to the general surgery service after presenting to the emergency department with acute epigastric abdominal pain for one-day duration. Radiography and a computed tomography (CT) scan of the abdomen revealed a large pneumoperitoneum. Subsequently, a diagnostic laparoscopy was performed, which detected a sealed perforation in the fundus of the wrapped-sleeved stomach, along with an incidental finding of intestinal malrotation. The encountered variation of anatomy created an intraoperative challenge during the conversion from Nissen-Sleeve gastrectomy to single anastomosis gastric bypass. The diagnosis of intestinal malrotation in adults is often overlooked, posing substantial diagnostic and management challenges when encountered.

## Introduction

Intestinal malrotation is a variation in the rotation or fixation of the gastrointestinal tract during fetal development. It results from a partial or complete failure of a 270-degree counterclockwise rotation during embryologic midgut development around the superior mesenteric artery [1].

Intestinal malrotation is considered a disease of infancy owing to the majority of cases presenting during the first year of life, 30% of cases present within the first month of life, 60% present within one year of age, and over 75% before the fifth year, with adult presentation comprising only 0.2-0.5% of all diagnosed cases [2-3].

Herein, we present a case of intestinal malrotation in a 48-year-old woman with a previous history of Nissen-sleeve gastrectomy presenting with a sealed gastric perforation noted during a diagnostic laparoscopy. This unexpectedly encountered variation posed challenges while undergoing a conversion to a single anastomosis gastric bypass due to the altered anatomy.

## Case presentation

A 48-year-old Bahraini woman with a pertinent history of dyslipidemia and gastroesophageal reflux disease who had undergone laparoscopic Nissen-sleeve gastrectomy before 11 months presented to the emergency department of another hospital with a complaint of sudden-onset epigastric pain associated with nausea and vomiting of gastric contents, which started one day prior to her presentation.

**Figure 1:**
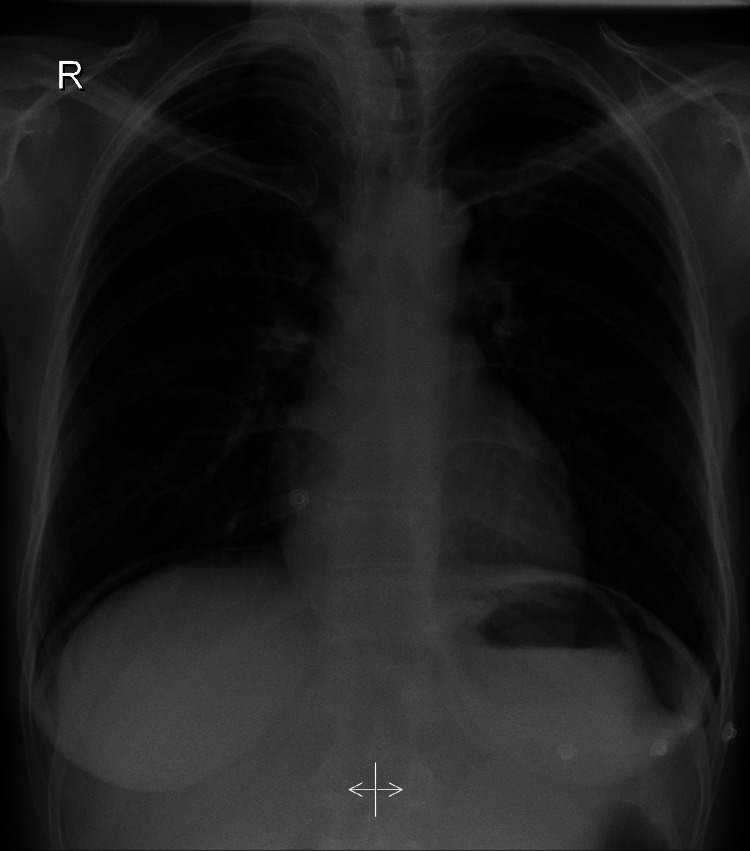
Chest radiograph showing air under the diaphragm bilaterally

Upon initial clinical presentation, the patient had stable vital signs (heart rate: 82 beats per minute, blood pressure: 147/84 mmHg, and temperature: 37.5 °C). Abdominal examination revealed minimal epigastric tenderness with no peritoneal signs. Further investigation included a CT of the abdomen, which revealed pneumoperitoneum without a clearly visible perforation. The patient was then referred to our institution for further management. Chest radiographs indicated the presence of air under the diaphragm bilaterally (Figure 1).

**Figure 2:**
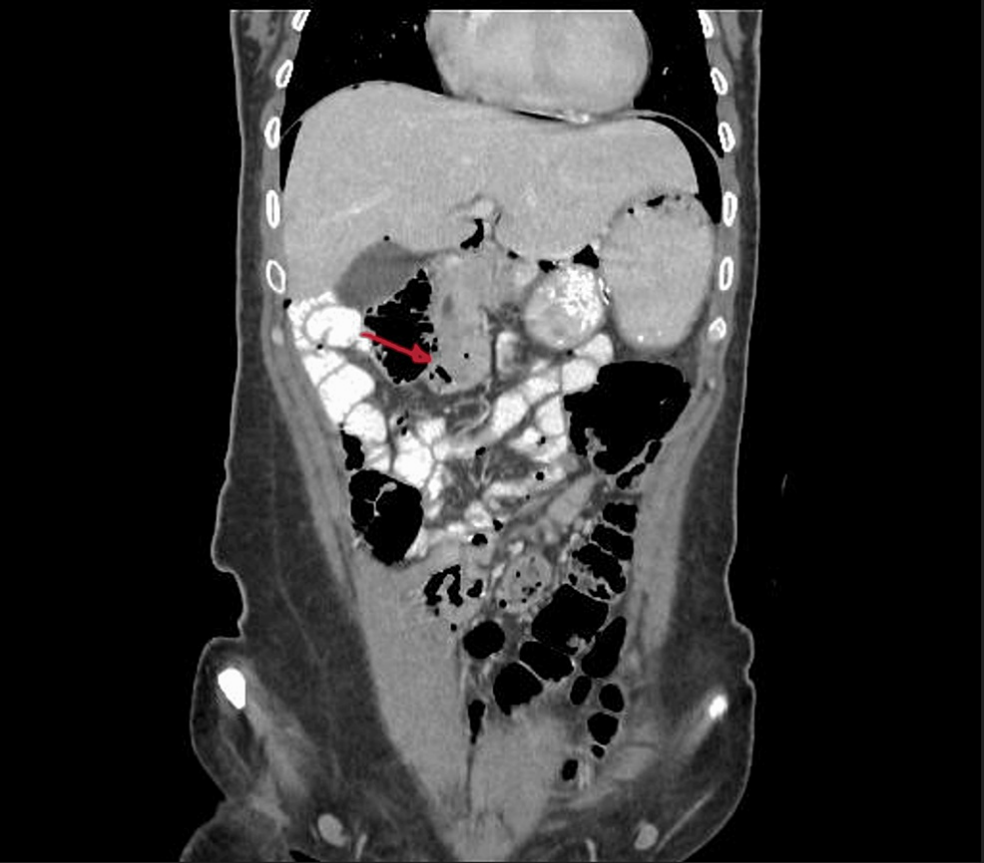
CT of the abdomen showing pneumoperitoneum and duodenojejunal junction (red arrow) observed on the right side of the abdomen

Abdominal CT showed a large amount of free intraperitoneal air, indicative of a perforated hollow viscus. The perforation could not be confidently identified; however, the location of the free air suggested a duodenal or stomach perforation. Notably, the duodenojejunal junction was observed on the right side of the abdomen (Figure 2).

A barium swallow study was performed, which showed no evidence of contrast leakage along the esophagus, gastroesophageal junction, or sleeved stomach; however, a considerable amount of pneumoperitoneum was observed. Based on the radiographic findings, the differential diagnosis included a sealed perforation in the stomach or duodenum. Owing to the patient’s stable clinical condition, further diagnostic investigations were planned rather than performing exploratory surgery.

After completion of diagnostic investigations, exploratory surgery was performed the following day, and the patient was taken to the operating theater and placed under general anesthesia in the supine position. An orogastric caliber tube was inserted after induction of anesthesia prior to surgery starting.

A supraumbilical horizontal incision of approximately 5 mm was made, and an Opti-Port was inserted under direct vision. Additional ports, including a 12 mm right upper abdominal port, a 12 mm left upper abdominal port, and a 5 mm epigastric port, were inserted under direct vision. A diagnostic laparoscopy of the peritoneal cavity was performed, which revealed the following findings: a minimal amount of free fluid in the left upper abdomen without frank contamination; an area of adhesions and exudate was observed in the gastric fundus, indicating the site of sealed perforation; and an acute angulation of the sleeved gastric pouch. The free fluid was suctioned, the pylorus was clamped using a straight bowel grasper, and methylene blue was injected into the orogastric tube until distension of the stomach was seen, with no leakage of methylene blue. The site of the sealed perforation was reinforced primarily with 2-0 polydioxanone (PDS) sutures, and an omental patch was applied.

**Figure 3:**
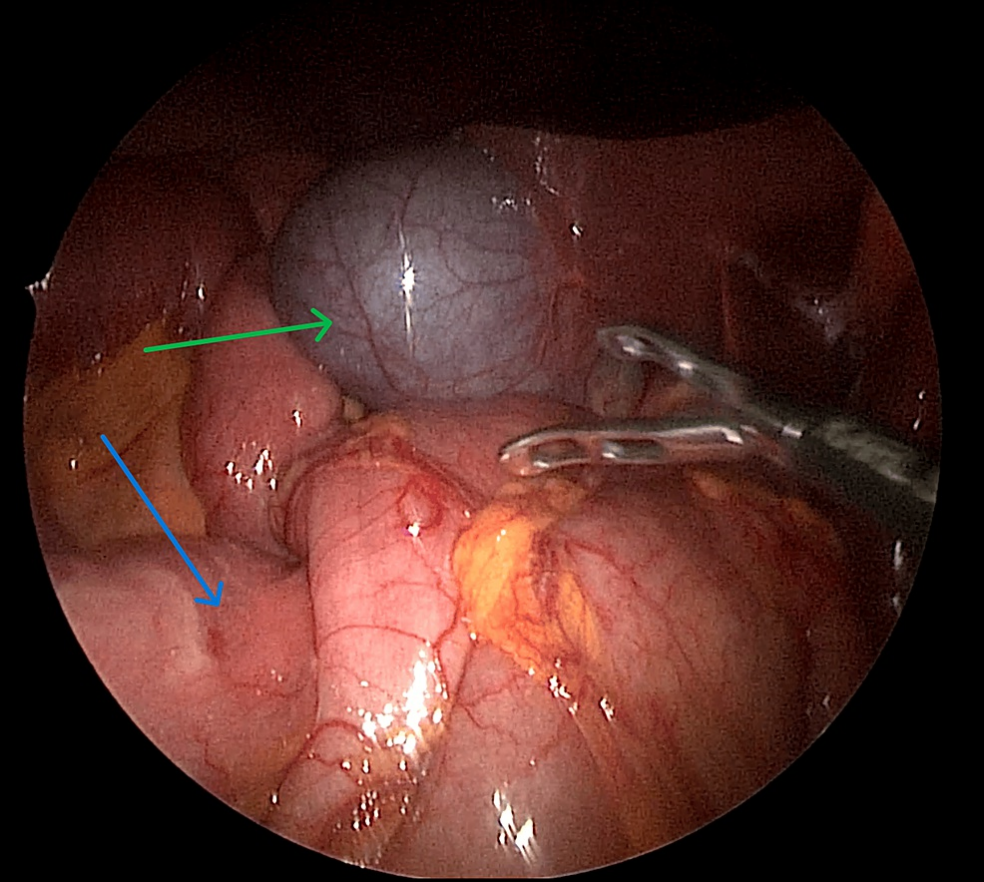
Intraoperative view showing the position of the duodenojejunal junction (blue arrow) in proximity to the gallbladder (green arrow) in the upper right quadrant of the abdomen

To overcome the increase in gastric pressure, a decision was made to convert the patient to a single anastomotic gastric bypass. A stomach pouch was created using an orogastric tube as a guide, up to the angle of His, separating the stomach pouch from the rest of the stomach. Interestingly, the duodenojejunal flexure was located on the right side of the abdomen (Figure 3). The entirety of the small bowel was located in the right hemi-abdomen with the ileocecal junction in the left hypochondrium, and the entirety of the large bowel was located in the left hemi-abdomen. A gastrojejunostomy was created with the newly formed gastric pouch at a distance of 100 cm distal to the duodenojejunal junction, and the newly created gastro-jejunal anastomosis was secured with 2-0 PDS sutures. A methylene blue leak test was performed, which indicated no leakage, and a 15-Fr Jackson-Pratt drain was inserted in the sub-hepatic space through the epigastric port. The patient remained clinically stable throughout the procedure, was shifted to the recovery room after the completion of the surgery, and was subsequently transferred to the general ward.

The patient was hospitalized for five days, and intravenous antibiotics were administered. A postoperative Gastrografin study was performed on the first postoperative day, which showed free flow of contrast to the small bowel with no leakage. The postoperative course was uneventful, and the patient was started on clear fluids on day one postoperatively. The epigastric drainage tube was removed on day five, and the patient was discharged in stable condition. Outpatient follow-up visits were scheduled for one week and one month postoperatively. The patient was healthy, with no postoperative complications, and had returned to her usual state of health.

## Discussion

**Figure 4:**
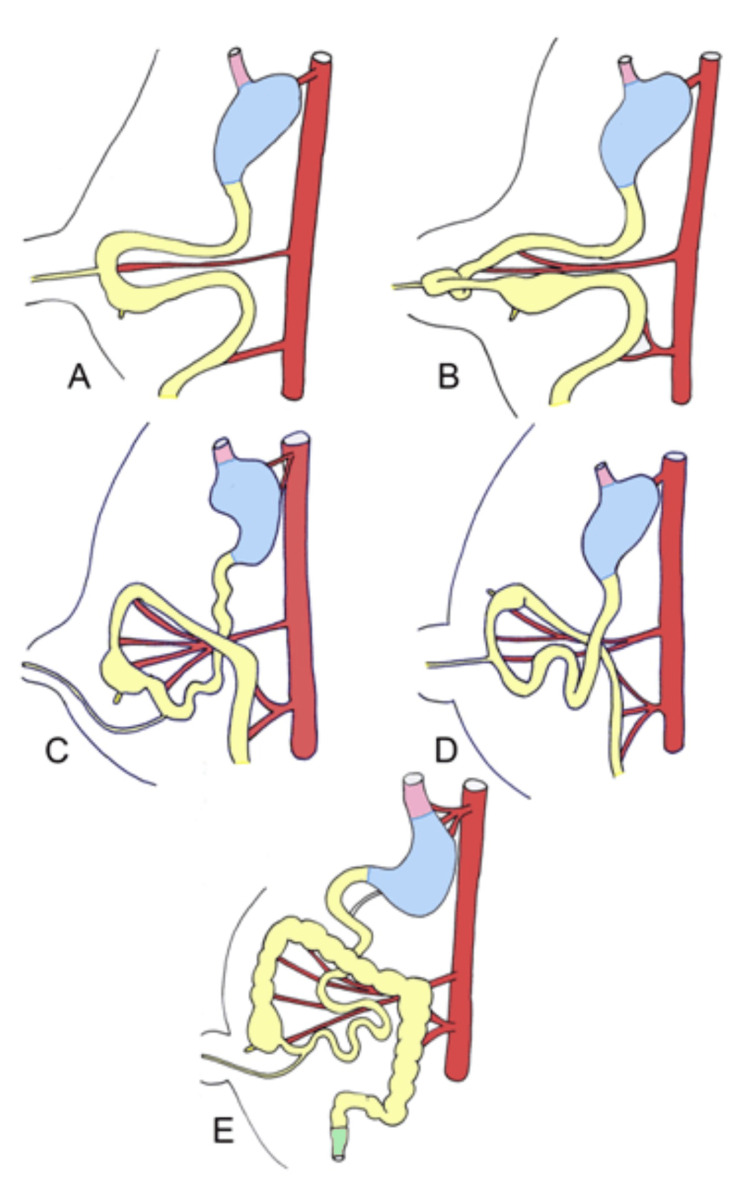
Illustration of the stages of intestinal development, herniation, and rotation of the mid-gut loop at 6–10 weeks, followed by the return of the mid-gut into the abdomen, fixation, and descent of the cecum to the right lower abdomen Image Credit: Danowitz and Solounias (2016) [4]

In normal embryologic development, the midgut prolongs and herniates out of the abdominal cavity, undergoing a 90° rotation around the superior mesenteric artery between weeks 5 and 10 of gestation (stage 1) (Figure 4A-4B). After the 10th week, the intestines re-enter the abdominal cavity (Figure 4C), undergoing a 180° rotation in a counterclockwise direction (stage 2) (Figure 4D), followed by the descent of the cecum into the right lower abdomen with fixation of the mesentery during the 11th week (stage 3) (Figure 4E) [4].

This 270° rotation leads to the normal orientation of the small and large intestines. Intestinal malrotation is classically defined as any variation in the abovementioned rotation or fixation of the intestines during development, which leads to a range of orientations depending on the stage at which normal development is altered, predisposing patients to several acute and chronic complications [5]. Volvulus, the most serious complication, can impair blood supply, potentially leading to necrosis and possibly sepsis.

In our case report, an intestinal malrotation was detected incidentally in an adult patient during a diagnostic laparoscopy. The greatest intraoperative challenge was the unexpected variability in anatomy encountered during the conversion from a Nissen-sleeve gastrectomy to a single anastomotic gastric bypass. Because such malformations can potentially impact surgical approaches and management, surgeons must be prepared to adapt and modify their strategies based on the intraoperative findings. Intestinal malrotation in adults is well documented in the medical literature [6], and its diagnosis is often overlooked, leading to diagnostic and surgical challenges.

The treatment for malrotation was first described by Ladd in 1932, who advocated a surgical approach for intestinal malrotation, currently known as Ladd’s procedure [7]. To date, the need for prophylactic surgical intervention for incidentally found malrotation in asymptomatic patients aged two years and older remains inconclusive [8]. Future research exploring the best practices for diagnosing and managing intestinal malrotation in adults is warranted to improve patient outcomes.

## Conclusions

Herein, we present a case of intestinal malrotation that was incidentally detected in an adult patient during an emergent diagnostic laparoscopy. Despite undergoing previous laparoscopic surgery and a thorough radiographic investigation, the diagnosis was not established. This unexpected intraoperative finding posed challenges in creating the gastrojejunal anastomosis due to the altered anatomy. This highlights the risk of incorrect anastomotic sites during conversion procedures if the malrotation is not identified.
